# Description of a new leafhopper species of the genus *Longicornus* (Hemiptera, Cicadellidae, Deltocephalinae) from China, with a revised key to species

**DOI:** 10.3897/zookeys.888.34799

**Published:** 2019-11-11

**Authors:** Yongqin Fang, Jichun Xing

**Affiliations:** 1 Institute of Entomology, Guizhou University, The Provincial Special Key Laboratory for Development and Utilization of Insect Resources, Guizhou University, Guiyang, 550025, China Guizhou University Guiyang China

**Keywords:** distribution, Homoptera, leafhopper, morphology, taxonomy

## Abstract

A new leafhopper species *Longicornus brevispinus***sp. nov.** is described and illustrated from Yunnan Province, China. A key to distinguish all species of this genus is given, and a map showing the geographic distribution of all species is also provided. The type specimen of the new species is deposited in the Institute of Entomology, Guizhou University, Guiyang, China.

## Introduction

Li and Song (2008) established the genus *Longicornus* with *L. flavipuncatus* Li & Song, 2008 as its type species from China. This genus belongs to the tribe Scaphoideini of the subfamily Deltocephalinae based on the head being narrower than the pronotum, the frontoclypeus long and narrow, antennae long, and the forewing with one or more darkly pigmented reflexed veins in the vicinity of the outer anteapical cell (Zahniser and Dietrich 2013). Recently, Fang and Xing (2018) reviewed this genus and added two new species: *L. furcatus* Fang & Xing and *L. biprocessus* Fang & Xing, and considered *L. flavipuncatus* Li & Song, 2008 as a senior synonym of *L. yunnanensis* Xing & Li, 2011. So far, this genus includes five species, all from China.

During a study of the Chinese Deltocephalinae, we discovered another new species *L. brevispinus* sp. nov. from Yunan Province, China, which is described here. A key is also given to separate all five species of the genus. The type specimen of the new species is deposited in the Institute of Entomology, Guizhou University, Guiyang, China (GUGC).

## Material and methods

Male specimens were used for the description and illustration. External morphology was observed under a stereoscopic microscope and characters were measured with an ocular micrometer. Color photographs were taken and stacked using a Nikon SMZ25 microscope. The genital segments of the specimens examined were macerated in 10% NaOH washed in distilled water and stored in glycerol. Male genital structures were drawn from preparations in glycerin jelly using a Leica MZ 12.5 stereomicroscope. Illustrations were scanned with a Canon CanoScan LiDE 200 and imported into Adobe Photoshop CS8 for labeling and plate composition.

Terminology of morphological and genital characters mainly follows Li et al. (2011) and Fang and Xing (2018). Absolute measurements, in millimeters (mm), are used for the body.

## Taxonomy

### Longicornus

Taxon classificationAnimaliaHemipteraCicadellidae

Li & Song

2977D661-00D2-5017-8863-1BA226C947E0

Longicornus Li & Song, 2008: 27; Li et al. 2011: 110; Zahniser and Dietrich 2013: 152; Fang and Xing 2018: 435.

#### Type species.

*Longicornus flavipuncatus* Li & Song, 2008.

#### Remarks.

For the relationship and diagnosis of *Longicornus* see Fang and Xing (2018: 436).

#### Distribution.

China (Guizhou, Sichuan, Yunnan).

##### Checklist of species of *Longicornus*

*L. biprocessus* Fang & Xing, 2018: 440, figs 10–12; 34–40. China (Sichuan).

*L. brevispinus* sp. nov., Figs 1–11. China (Yunnan).

*L. flavipuncatus* Li & Song, 2008: 28, figs 1–8. China (Sichuan, Guizhou, Yunnan).

*L. yunnanensis* Xing & Li, 2011: 112, figs 5–102: 1–8 (in Li et al. 2011), synonymized by Fang and Xing 2018: 436.

*L. furcatus* Fang & Xing, 2018: 439, figs 7–9; 27–33. China (Sichuan).

*L. longus* Xing & Li, 2011: 112, figs 5–101: 1–7. China (Yunnan).

##### Key to species (males) of Longicornus*

**Table:** 

1	Aedeagal shaft with a pair of processes arising apically (Figs 8, 9; Fang and Xing 2018: figs 16,17, 23, 24)	**2**
–	Aedeagal shaft with pair of processes arising basally (Fang and Xing 2018: figs 30, 31, 37, 38)	**4**
2	Aedeagal shaft processes longer than shaft (Fang and Xing 2018: figs 16, 17)	***L. flavipuncatus***
–	Aedeagal shaft processes shorter than shaft (Figs 8, 9; Fang and Xing 2018: figs 23, 24)	**3**
3	Aedeagus long, and its apical processes approximately ¾ as long as shaft (Fang and Xing 2018: figs 23, 24)	***L. longus***
–	Aedeagus short and stout, and its apical processes shorter than half length of aedeagal shaft (Figs 8, 9)	***L. brevispinus* sp. nov.**
4	Aedeagal shaft with pair of furcate processes arising from ventral margin near base (Fang and Xing 2018: figs 30, 31)	***L. furcatus***
–	Aedeagal shaft with two pairs of processes medially on dorsal margin (Fang and Xing 2018: figs 37, 38)	***L. biprocessus***

### Longicornus brevispinus sp. nov.

Taxon classificationAnimaliaHemipteraCicadellidae

E651E7AF-6D7F-5183-8628-87D5CE9103B4

http://zoobank.org/267A75A3-4AEE-4D63-9F63-F117D095674D

Figs 1–4 , 5–11

#### Description.

**Figures 1–4.**
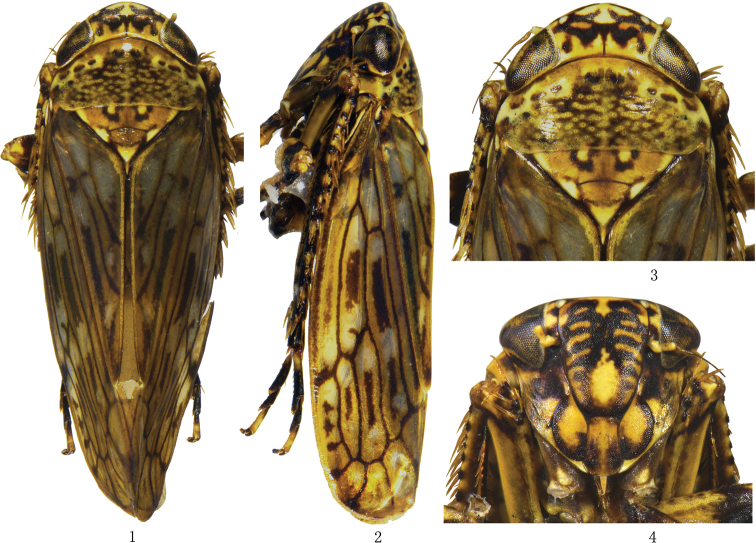
*Longicornus brevispinus* sp. nov., **1** ♂, dorsal view **2** ♂, lateral view **3** ♂, head and thorax, dorsal view **4** ♂, face, ventral view.

Body robust, yellowish brown (Figs 1–4). Vertex with paired irregular dark brown short coalescing bands. Eyes black, ocelli pale yellow. Face marked with dark brown. Pronotum with irregular fuscous patches. Forewing brownish, with scattered hyaline areas, veins dark brown. Legs dark brown.

Head including eyes slightly narrower than pronotum. Vertex with fore margin produced roundly, median length distinctly shorter than width between eyes. Ocelli located on anterior margin of vertex. Frontoclypeus distinctly longer than wide, anteclypeus expanded apically. Antennae arising near lower corner of eye. Pronotum with anterior margin roundly produced and posterior margin concave, longer than vertex. Mesonotum triangular, slightly shorter than pronotum, with transverse suture depressed. Forewing with four apical and three subapical cells, about 3 times as long as wide, appendix wide. Hind wing with three apical cells and two anteapical cells. Fore femur row IC with a row of short setae, row AM with 1 stout seta, 2 dorsoapical setae, and row AV with several short setae in basal half. Fore tibia with 4 macrosetae in row AD and numerous macrosetae decreasing in length toward the base in row AV. Hind femur broadened distally and slightly bowed, apical setal formula 2 + 2 + 1. Hind tibia flattened and nearly straight, row PD with 12 macrosetae decreasing in length toward the base; row AD with 10 long stout setae and 1–4 shorter stout setae between each long seta; metabasitarsomere with 4 platellae and 2 setae on apical transverse row.

**Figures 5–11.**
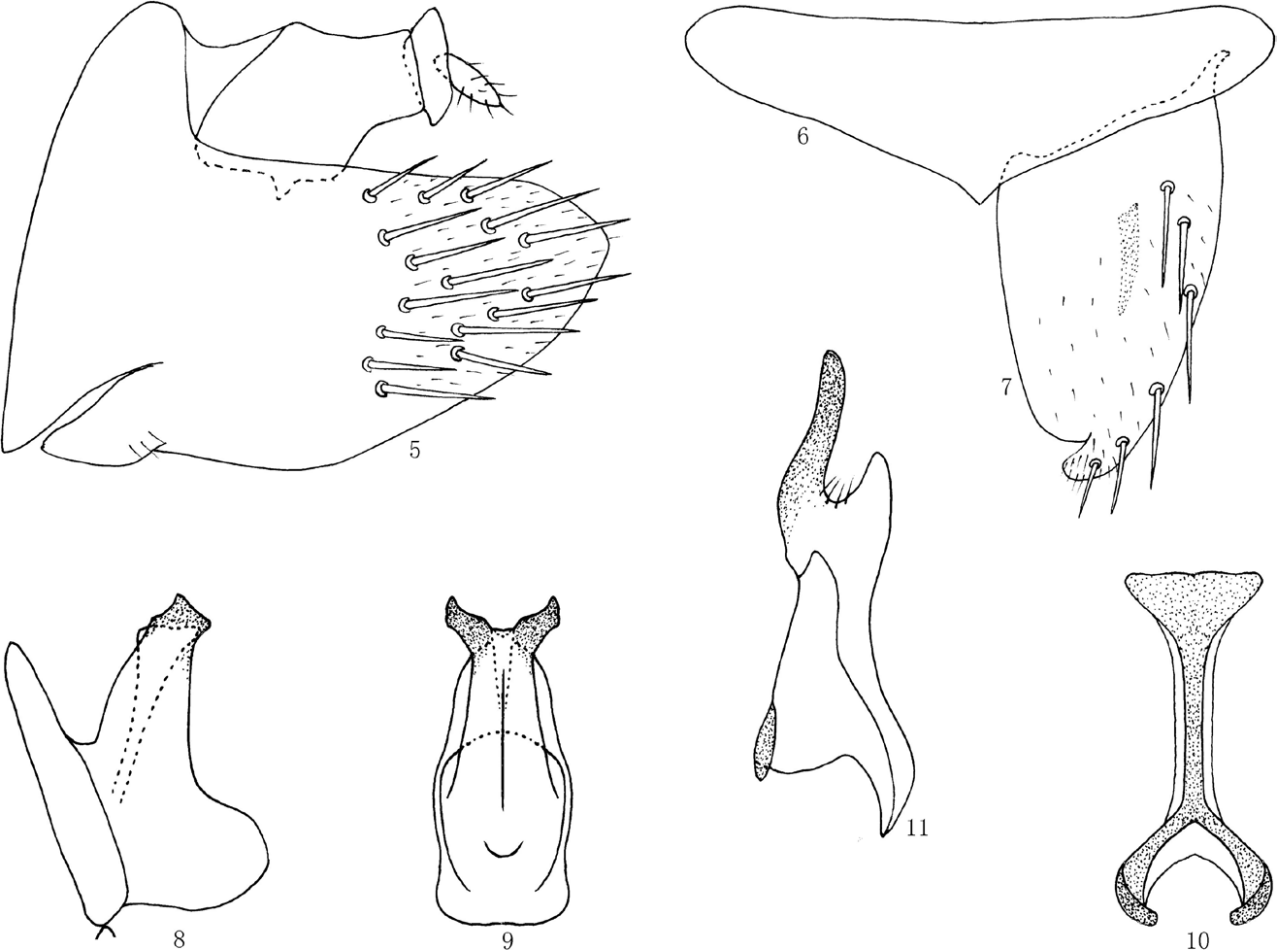
*Longicornus brevispinus* sp. nov., **5** male pygofer side, lateral view **6** valve, ventral view **7** subgenital plate, ventral view **8** aedeagus, lateral view **9** aedeagus, ventral view **10** connective, dorsal view **11** style, dorsal view.

***Male genitalia***: Pygofer longer than broad in lateral view, with many long macrosetae in posterior half (Fig. 5). Valve subtriangular (Fig. 6). Subgenital plate with wide base, narrowed posteriorly, with 6 setae along lateral margin, and mesal margin deeply concave near apex (Fig. 7). Style relatively narrow, apical process acute, turned laterally (Fig. 11). Connective articulated with aedeagus, Y-shaped with stem long (Fig. 10). Aedeagus very short and stout with base broad in lateral view, gradually tapered to apex in lateral view, with a pair of short and robust apical processes with truncate apex, gonopore apical (Figs 8, 9).

#### Measurement.

Length (including tegmen): ♂, 5.6 mm.

#### Type material.

***Holotype*** ♂, China: Yunnan Prov., Pingbian country, Daweishan, 22 May 2015, coll. Jiajia Wang (GUGC).

#### Distribution.

China (Yunnan).

#### Remarks.

The new species can be distinguished by the very short and stout aedeagus with a pair of short and robust apical processes with a truncate apex.

#### Etymology.

The species name is derived from the Latin word “*brevis*” and “*spinus*”, referring to the short apical processes of the aedeagal shaft.

## Discussion

Species of *Longicornus* are all very similar in coloration and difficult to distinguish externally, but the structure of aedeagus are markedly different. This genus now includes five species which can be divided into two types based on the structure of aedeagus: 1) aedeagus with one pair of apical processes (*L. brevispinus* sp. nov., *L. flavipuncatus* and *L. longus*); 2) aedeagus with paired basal processes (*L. furcatus* and *L. biprocessus*). *Longicornus furcatus* has one pair of furcate aedeagal processes arising from the ventral margin near the base and *L. biprocessus* has two pairs of aedeagal processes medially on the dorsal margin of the shaft.

**Figure 12.**
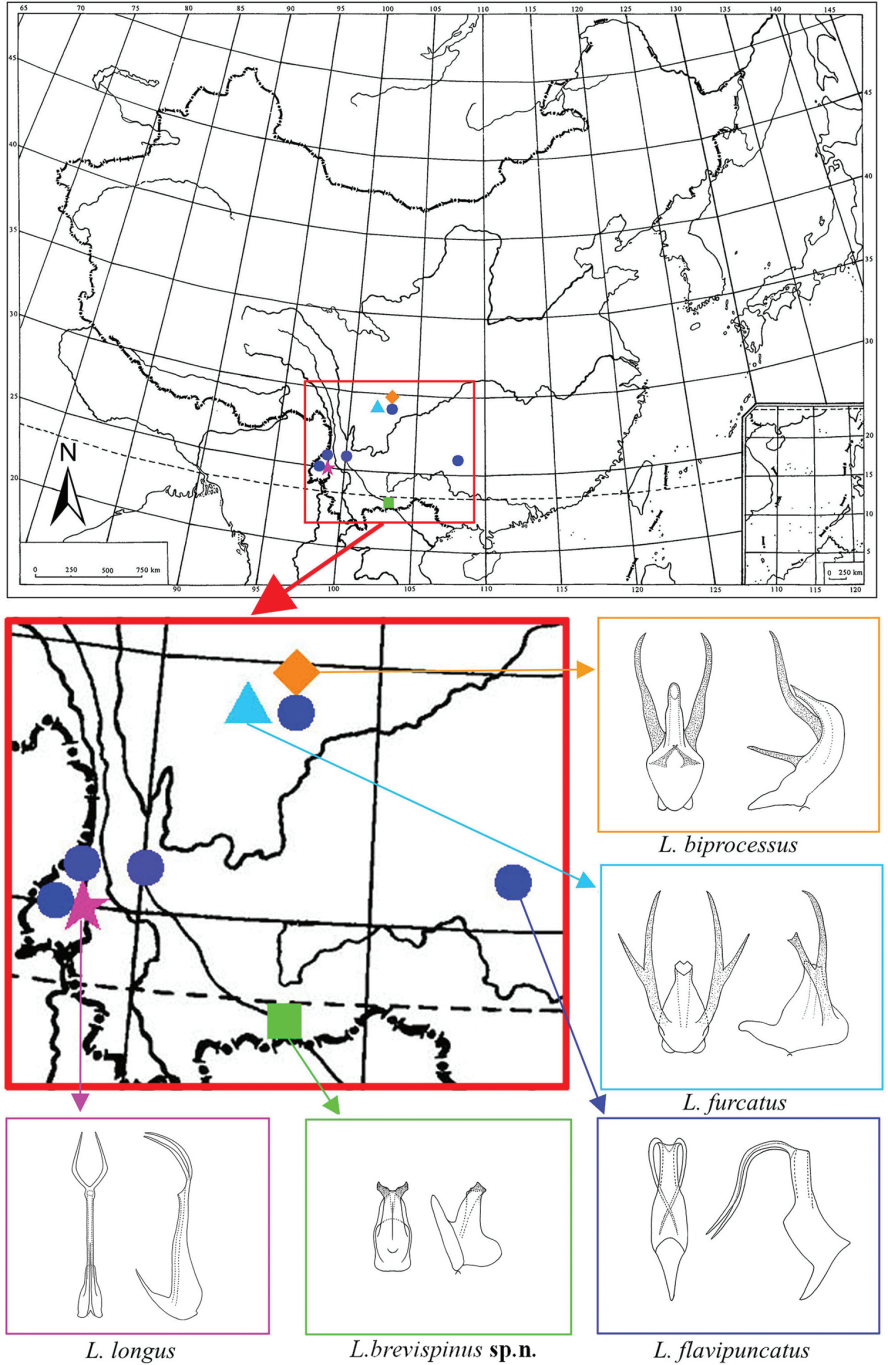
Geographic distribution of *Longicornus* species.

All species of *Longicornus* are distributed in southwest China (Oriental Region) and the species without apical processes of the aedeagus are distributed in the north of the region (Fig. 12). So far, this genus has not been recorded in the Palaearctic Region of China but it is highly likely that undiscovered species may be found there.

## Supplementary Material

XML Treatment for Longicornus

XML Treatment for Longicornus brevispinus

## Footnotes

* Modified from Fang and Xing 2018
